# Modulation of inflammation by autophagy: Consequences for human disease

**DOI:** 10.1080/15548627.2015.1071759

**Published:** 2015-07-29

**Authors:** Romana T. Netea-Maier, Theo S. Plantinga, Frank L. van de Veerdonk, Johannes W. Smit, Mihai G. Netea

**Affiliations:** a Department of Internal Medicine, Radboud University Medical Center, Nijmegen, The Netherlands; b Division of Endocrinology, Radboud University Medical Center, Nijmegen, The Netherlands; c Radboud Center for Infectious Diseases, Radboud University Medical Center, Nijmegen, The Netherlands

**Keywords:** autoimmune diseases, autoinflammatory diseases, autophagy, carcinogenesis, infectious diseases, inflammation

## Abstract

Autophagy and inflammation are 2 fundamental biological processes involved in both physiological and pathological conditions. Through its crucial role in maintaining cellular homeostasis, autophagy is involved in modulation of cell metabolism, cell survival, and host defense. Defective autophagy is associated with pathological conditions such as cancer, autoimmune disease, neurodegenerative disease, and senescence. Inflammation represents a crucial line of defense against microorganisms and other pathogens, and there is increasing evidence that autophagy has important effects on the induction and modulation of the inflammatory reaction; understanding the balance between these 2 processes may point to important possibilities for therapeutic targeting. This review focuses on the crosstalk between autophagy and inflammation as an emerging field with major implications for understanding the host defense on the one hand, and for the pathogenesis and treatment of immune-mediated diseases on the other hand.

## Introduction

Autophagy is the physiological cellular process through which intracellular components undergo lysosome-mediated self-digestion and recycling.^1^ In conditions of cellular stress such as starvation, hypoxia, or exposure to toxic molecules, the autophagy machinery is activated in order to maintain cell homeostasis. An increasing body of evidence has emerged supporting the view that autophagy is involved in several physiological processes including cell metabolism, cell survival, and host defense.^2^ It can be argued that autophagy is a primordial form of eukaryotic innate immunity against invading microorganisms. Research performed in the past decade has also proposed that, in mammals, these primordial functions of autophagy have evolved and now encompass multiple innate and adaptive immune mechanisms.^3^ Moreover, defective autophagy is associated with several pathological conditions such as cancer, autoimmune disease, neurodegenerative disease, and senescence.^4^ These new insights strongly suggest that besides its classical role as a “housekeeping” mechanism, autophagy can also be considered as crucial for host defense in general, and regulation of inflammation in particular. In this review we present the newest concepts that link these 2 fundamental biological processes, autophagy, and inflammation.

## Autophagy and host defense

**Figure 1.**
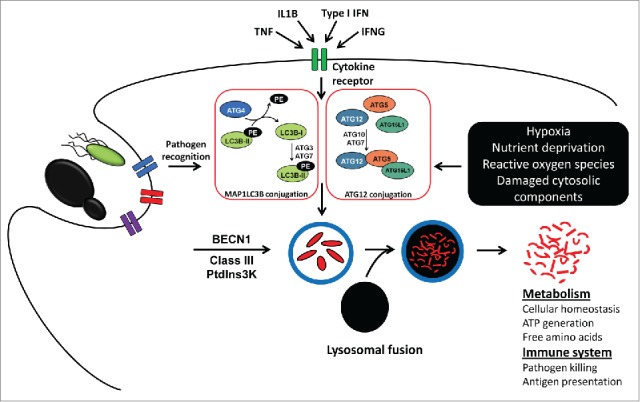
The process of autophagy in eukaryotic cells and its involvement in cellular homeostasis, metabolism, and the immune system. Autophagy is induced both by metabolic and immune signals comprising pathogen recognition and stimulation by proinflammatory cytokines. Upon autophagy triggering through any of these pathways, the MAP1LC3B (depicted as unconjugated LC3B-I and PE-conjugated LC3B-II) and ATG12 conjugation systems, as well as BECN1 and class III PtdIns3K, are activated that are critical for autophagosome formation. After engulfment of the autophagic cargo, autophagosomes proceed to lysosomal fusion, leading to degradation of the sequestered content. In case the autophagic cargo originates from intracellular organelles or protein complexes, the degraded fragments are utilized for metabolic purposes to generate ATP and provide the cell with free amino acids. In contrast, autophagy-mediated degradation of extracellular material, especially microbes, enables pathogen killing and activation of adaptive immunity by MHC-dependent antigen presentation.

Several types of autophagy have been recognized, including macroautophagy, microautophagy, chaperone-mediated autophagy, and noncanonical autophagy.^5^ In this review we will focus on macroautophagy, which will be henceforth referred to as autophagy. This process is activated in response to a multitude of stress factors including starvation, hypoxia, and other toxins that result in accumulation of damaged organelles and protein aggregates. As a consequence, a double-layer membrane, the phagophore, is formed to engulf the damaged cytosolic components (Fig. 1). After complete sequestration of these components, the resulting autophagosome fuses with the lysosome, which enables the breakdown of the autophagosome inner membrane and exposes the cargo to the lysosomal hydrolases. The resulting breakdown products are subsequently released in the cytosol and are available to be reused for the synthesis of required macromolecules.^5^ Several autophagy-related (*ATG*) genes and proteins are involved in the induction of autophagy (Fig. 1), while a subset of them also have autophagy-independent functions.^6^

Among the most important functions of autophagy in addition to maintaining cell homeostasis is its role in host defense. Autophagy improves host defense mechanisms through several biological functions: the direct elimination of the invading pathogens (involving also xenophagy and MAP1LC3/LC3 [microtubule-associated protein 1 light chain 3]-associated phagocytosis-LAP),^7^ control of adaptive immunity through regulation of antigen handling and presentation,^8,9^ induction of innate immune memory or trained immunity,^10^ and modulation of inflammation. Indeed, autophagy has been suggested to represent an ancient form of innate immune response to an infection.^3^ Engagement of various families of pattern-recognition receptors (PRRs) has been reported to induce autophagy through pathways that are similar to those activated in response to nutrient deprivation, and mediated via MTOR (mechanistic target of rapamycin [serine/threonine kinase]) and adenosine monophosphate (AMP)-activated protein kinase (AMPK). In this respect, engagement of TLR4 (toll-like receptor 4) by lipopolysaccharide (LPS) of Gram-negative bacteria results in the ubiquitination of BECN1/beclin-1 by TRAF6 (TNF receptor-associated factor 6, E3 ubiquitin protein ligase).^11^ TRAF6 also activates the serine/threonine protein kinase ULK1 (unc-51 like autophagy activating kinase 1), both pathways leading to the activation of autophagy.^12^ Interestingly, the LPS-induced autophagy is dependent on MAPK/p38 and TICAM1/TRIF (toll-like receptor adaptor molecule 1), but not on MYD88 (myeloid differentiation primary response 88).^13^ Similarly, the engagement of several other TLRs also induces autophagy.^14^ Not only TLRs, but nucleotide-binding oligomerization domain (NOD)-like receptors (NLRs) as well have been demonstrated to induce autophagy: NOD2 induces autophagy in dendritic cells and directs antigen handling and presentation,^15^ a process that involves targeting of ATG16L1 to the plasma membrane at the site of bacterial entry.^16^

In addition to PRRs, several proinflammatory cytokines including TNF (tumor necrosis factor)^17^ and IL1B (interleukin 1, β)^18^ induce autophagy and thus improve the control of infection. Furthermore, both type I (IFNA/IFNα-IFNB1/IFNβ-IL6) and type II (IFNG/IFNγ) interferons are major factors in activating autophagy.^19^ For instance, activation of macrophages by IFNG is crucial for intracellular killing of *Mycobacterium tuberculosis*,^20,21^ and type I IFN for host defense against viruses.^22^ In mice, IFNG induces the expression of autophagic p47 immunity-related guanosine-5′-triphosphate (GTP)ases (IRG) proteins,^21^ with IRGM1 (immunity-related GTPase family M member 1) being an important factor for inducing antimycobacterial autophagy. These findings are corroborated by the genetic association of *IRGM* polymorphisms with tuberculosis in West Africans, and increased Bacillus Calmette-Guérin (BCG) vaccination efficacy through enhanced antigen presentation.^23-25^ In contrast to the proinflammatory cytokines, T helper 2 (Th2) type responses and secretion of anti-inflammatory cytokines such as IL4 and IL13 antagonize autophagy induction through activation of MTOR.^26^ Altogether, the T helper 1/T helper 2 (Th1/Th2) responses seem to be correlated to the activation of autophagy, with autophagy being induced in Th1 type responses and inhibited when Th2 responses are elicited.

In addition, it must be underlined that some infectious agents are able to manipulate and inhibit autophagy, a *de facto* mechanism of evasion from the host defense. In line with this, human immunodeficiency virus (HIV) infection of CD4 lymphocytes reduces their content of BECN1 and MAP1LC3B-II, 2 crucial components for the induction of autophagy.^27^ Moreover, *M. tuberculosis* is also able to inhibit autophagy and phagolysosome biogenesis, a process that greatly contributes to the escape from the bactericidal mechanisms of the host.^28^ The fact that autophagy was targeted by microorganisms during evolution, with inhibitory effects increasing survival of the pathogens, demonstrates its importance for host defense.

In this review we will further focus on the modulatory effects of autophagy on inflammation, a broad concept with important consequences for the pathogenesis not only of infections, but of autoinflammatory diseases and malignancies as well.^3^

## The impact of autophagy on inflammation

Innate immunity represents the first line of defense against pathogens. It requires adequate pathogen recognition, which triggers activation of inflammation resulting in recruitment of immune cells, such as phagocytes, secretion of cytokines and chemokines, which in turn help recruit other immune cells and prime dendritic cells (DCs) for activation of adaptive immune responses. An increasing number of studies have recently shown that autophagy has important effects on the induction of the inflammatory reaction. However, when the inflammatory process is not properly controlled, autophagy can be detrimental for the host. In atherosclerosis, excessively stimulated autophagy can lead to endothelial cell death that can contribute to plaque destabilization, maintaining the inflammatory status of the plaque.^29^ In chronic obstructive pulmonary disease (COPD) cigarette smoking triggers a form of autophagy, namely mitophagy (autophagy-dependent selective elimination of mitochondria), through stabilization of the mitophagy regulator PINK1 (PTEN-induced putative kinase 1). This eventually leads to epithelial cell death, and thus can contribute to persisting inflammatory responses in COPD.^30^

## Autophagy effects on inflammasome activation and IL1B production

**Figure 2.**
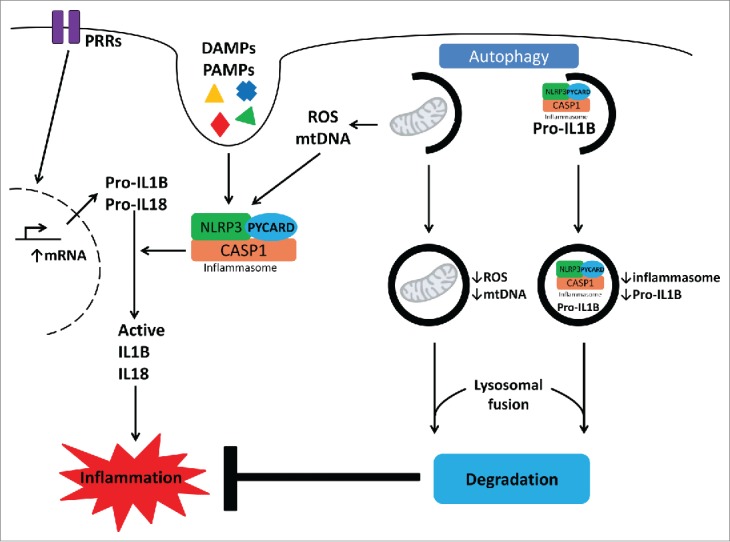
The interplay between autophagy and IL1B-inflammasome activation. Production of the pro-inflammatory cytokines IL1B and IL18 is pivotal in antimicrobial host defense, since this leads to activation of both innate and adaptive immune responses including Th1 and Th17. Autophagy potently regulates these immune responses by several means. First, autophagy inhibits IL1B and IL18 production through digestion of dysfunctional mitochondria and thereby prevention of mitochondrial ROS release that is known to activate the inflammasome. Second, autophagy is capable of targeting inflammasome complexes for degradation, which prevents cleavage of pro-IL1B and pro-IL18 into their biologically active counterparts. Finally, the autophagy machinery engulfs and eradicates pro-IL1B proteins, representing another level of IL1B regulation. DAMP, danger-associated molecular pattern; PAMP, pathogen-associated molecular pattern; PRR, pattern recognition receptor; ROS, reactive oxygen species.

Production of cytokines from the IL1 family, and especially IL1B, is one of the main mechanisms through which innate and adaptive immune responses are induced by an infection. The regulation of the production of this cytokine is thus crucial for understanding inflammation and host defense. Probably the best studied aspect of the interaction between autophagy and inflammation is represented by the effects of autophagy on inflammasome activation and IL1B release. An overview of the interplay between autophagy and IL1B-inflammasome activation is depicted in Figure 2. The inflammasome consists of protein complexes formed by one or more members of the NLR family of receptors together with CASP1/caspase-1, leading to the activation of this cysteine protease, and processing of the inactive pro-IL1B and pro-IL18 into active cytokines.^31^ The first observation regarding the effect of autophagy on inflammasome activation was that of Saitoh et al. who reported that macrophages of *Atg16l1* knockout mice respond with increased production of IL1B after stimulation with LPS. This was due to an exaggerated activation of CASP1 in the *Atg16l1*-deficient mice, mediated by the adapter molecule TICAM.^32^ Additional studies support the concept that autophagy regulates inflammasome activation.^33-36^

Several mechanisms have been proposed to mediate these anti-inflammatory effects of autophagy. During homeostatic conditions autophagy is responsible for the clearing of the cytoplasm of nonfunctional mitochondria and other organelles. If autophagy is defective this leads to an accumulation of depolarized mitochondria, that release inflammasome activators such as reactive oxygen species (ROS) or mitochondrial DNA (mtDNA).^33,34,37^ Moreover, autophagy may also remove aggregated inflammasome structures, thus contributing to dampening proinflammatory responses.^36^ These processes might play an important role during infections. This is underscored by the observation that influenza virus triggers RIPK2 (receptor-interacting serine-threonine kinase 2) to activate ULK1, to enhance mitophagy and control inflammasome activation.^38^ These studies in mice point toward a regulatory effect of autophagy on CASP1 activation through modulation of the NLRP3 (NLR family, pyrin domain containing 3) inflammasome.

The important role of autophagy for IL1B secretion was also confirmed in human primary cells, in which inhibition of autophagy leads to increased production of IL1B.^39^ Interestingly, the same study in humans demonstrated that TNF production is decreased by autophagy inhibition, suggesting divergent effects of autophagy on the production of these cytokines.^39^ However, important differences may exist between mice and humans regarding the effect of autophagy on IL1B production. In mice, this effect is ascribed to inhibition of inflammasome activation by autophagy, whereas in humans *IL1B* mRNA transcription is elevated when autophagy is inhibited, whereas no effects are observed on CASP1 activation.^39,40^ One potential mechanism for this observation was proposed to be due to targeting pro-IL1B for degradation,^40^ while a recent study also reported autophagy-dependent degradation of the PELI (pellino 3 ubiquitin protein ligase) with subsequent inhibition of IL1B expression during TLR4 signaling.^41^ These discrepancies between mice and humans emphasize that caution should be exercised when extrapolating studies from mice to humans. Moreover, it has recently been demonstrated that a noncanonical secretion pathway controlled by autophagy plays an important role in the secretion of proteins that lack a signal sequence, such as IL1B. In contrast to basal autophagy, which inhibits IL1B secretion,^33^ induced autophagy augments IL1B secretion via an unconventional secretion mechanism that utilizes the autophagic machinery.^35^

## The effect of autophagy on other inflammatory pathways

Autophagy does not only have inhibitory effects on inflammasome activation, but also on inflammatory mediators that are independent of CASP1 activation. In line with this, autophagy suppresses CAPN/calpain-dependent IL1B activation, and this effect is linked to the release of ROS as well.^42^ In addition, autophagy reduces NFKB (nuclear factor of kappa light polypeptide gene enhancer in B-cells) activation by selective degradation of BCL10 complexes.^43^ This process is mediated through NSFL1C (NSFL1 [p97] cofactor [p47]), a negative regulator of IKBKB/IKK acting via degradation of the polyubiquitinated NFKB modulator IKBKG/NEMO.^44^

In addition to these effects on proinflammatory cytokines, autophagy also amplifies TLR signaling to improve the delivery of microbial ligands to cytoplasmic receptors such as TLR7 that promote the synthesis of type I IFN.^45^ This could also lead, however, to inappropriate immune activation, as shown by the increased trafficking of DNA-containing antigens and increased IFNA secretion.^7,46^ However, the effects of autophagy on the stimulation of type I IFN is complex, with the ATG12–ATG5 complex inhibiting the signaling induced by RIG-I-helicase like receptors (RLRs) by direct binding to the caspase-recruitment domain of the RLR adaptor molecule MAVS.^47^

These data suggest therefore that inflammation and autophagy are intertwined processes during host defense, and derangements in the crosstalk between these 2 processes can have important consequences for the pathogenesis and treatment of several diseases.

## Autophagy in the pathogenesis and treatment of immune-mediated disease

Due to the mechanisms described above, autophagy has emerged as a central player in the pathogenesis of immune-mediated diseases, including autoinflammatory and autoimmune diseases, infections, and cancer.

## The impact of autophagy on autoinflammatory and autoimmune diseases

**Figure 3.**
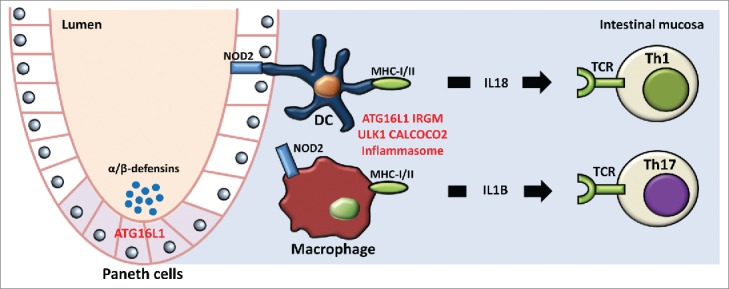
Interplay between autophagy and inflammation in the pathogenesis of Crohn disease. Multiple facets of inflammatory pathways involved in CD pathogenesis are influenced by autophagy, ranging on the one hand from defective microbial recognition and phagocytosis in dendritic cells (DCs) through the PRR NOD2, diminished antigen processing and presentation on MHC class I/II caused by genetic variants in ATG16L1, IRGM, and CALCOCO2, and on the other hand to a loss of inhibition of IL1B and IL18 production, important pro-inflammatory mediators leading to generation of Th1 and Th17 responses. Furthermore, genetic variation in ATG16L1 impairs the capacity of Paneth cells to excrete antimicrobial peptides, including DEFA/α- and DEFB/β-defensins. All together, these defects allow for microbial overgrowth and lead to hyperinflammation. DC, dendritic cell; TCR, T cell receptor.

The first major autoinflammatory disease in which autophagy has been suggested to play a significant role is Crohn disease (CD). CD is a chronic inflammatory disorder of the intestine caused by a combination of multiple factors including defective host response, altered mucosal barrier function, and exaggerated cytokine production. Genetic variants of important autophagy genes such as *ATG16L1, IRGM*, and *ULK1*, have been reproducibly associated with susceptibility and clinical outcome of the disease, demonstrating the important role of autophagy in CD.^48-51^ Functional studies have revealed that autophagy and subsequently inflammatory pathways are heavily influenced by genetic variation in autophagy genes, the *ATG16L1* variant in particular, as has been demonstrated for defective Paneth cell function,^52^ impaired bacterial defense,^53,54^ aberrant antigen presentation,^55,56^ and increased production of proinflammatory cytokines including IL1B and IL18.^57-59^ The *IRGM* promoter polymorphism risk allele influences IRGM expression, at least in part through posttranscriptional regulation by miRNAs.^60,61^ Furthermore, a well-established genetic risk factor with the highest predictive value for CD is for the gene encoding NOD2, a PRR that recognizes peptidoglycans and activates immune pathways and autophagy.^62-65^ Another indication that autophagy is a central process in intestinal homeostasis is the recent finding of *CALCOCO2/NDP52* mutations in CD patients, a gene that codes for an important factor in phagophore targeting of intracellular bacteria (Fig. 3).^66,67^

In addition to CD, important insights have also been provided by the assessment of autophagy induction and inflammasome activation in patients with chronic granulomatous diseases (CGD). CGD is a primary immunodeficiency characterized by defective ROS production due to mutations in the proteins forming the NOX (NADPH oxidase) complex.^68^ In addition to the defects in host defense, CGD is characterized by the presence of hyperinflammatory features, such as colitis that is indistinguishable from CD in 30% of the patients.^69^ Earlier studies have demonstrated that IL1B production capacity of monocytes and macrophages isolated from CGD patients is increased compared to controls,^70,71^ bringing into question the importance of NADPH-dependent ROS production for inflammasome activation. Since NADPH-oxidase dependent ROS was shown to be important for the induction of LAP,^72^ this led to the hypothesis that defective LAP in CGD may lead to hyperinflammation. Indeed, a recent study showed defective LAP in both patients with CGD and mice with mutations in genes encoding the NOX complex, which contribute to the exaggerated IL1B production.^73^ More importantly, treatment of CGD patients having colitis with recombinant IL1RN/IL1RA (interleukin 1 receptor antagonist) significantly improve the clinical picture.^73,74^ IL1RN not only reduces excessive IL1-driven inflammation, but also restores deficient LAP in NOX-deficient cells. IL1RN also has intracellular effects, and cellular uptake of IL1RN has been reported.^75^ Therefore, IL1RN might influence LAP through several possible mechanisms: i) direct inhibition of IL1 bioactivity, ii) intracellular interaction with the LAP machinery or iii) indirectly via effects on other processes such as inflammasome activation which is responsible for pro-IL1B processing.

Epidemiological studies assessing genetic variants in autophagy genes demonstrated their association with susceptibility to autoimmune disease as well, such as variants in *ATG5* associated with systemic lupus erythematosus (SLE)^76-78^ and systemic sclerosis.^79^ Furthermore, ATG5 expression is elevated in both blood and brain-residing T-cells in multiple sclerosis patients and is associated with the relapsing-remitting disease phenotype.^80^ These *ATG5* genetic variants are functionally linked to a differential *ATG5* expression and exhibit gene-gene interactions with *ATG7* and *IRGM*,^81^ thereby likely influencing the degree of activation of the autophagy process. Interestingly, ATG5 also plays an important role in clearing apoptotic cells,^82^ and a hallmark of SLE is the deficiency to clear apoptotic cells. Similar to CD, IRGM has also been implicated in multiple sclerosis based on the observation that IRGM expression is strongly induced in affected lesions of multiple sclerosis patients. Consistently, IRGM1-deficient mice are resistant to the experimental model of multiple sclerosis, antigen-induced experimental autoimmune encephalomyelitis (EAE), by promoting blood-brain barrier integrity and preventing T cell infiltration into the central nervous system.^83,84^

SLE is characterized by deregulation of autoantibody-producing B cells, infiltration of target organs by inflammatory T cells and aberrant immune cell activation. The latter is caused by abnormal activation of myeloid cells triggered by the presence of immune complexes composed of self-DNA from apoptotic cells and antibodies generated by autoreactive B cells.^85,86^ While T cells are considered as a major cell type driving initiation and progression of SLE,^87,88^ innate immune cells are also implicated in the disease process. In this respect, inappropriate handling of apoptotic bodies by phagocytes leads to accumulation of autoantigens that could promote loss of tolerance, predisposing the organism to autoimmune responses.^89,90^ Autophagy is likely involved in these processes, since it influences T cell development and activation, but also because it executes clearance of apoptotic cells and contributes to the elimination of their immunogenic potential.^91^

In rheumatoid arthritis (RA), a chronic inflammatory disorder affecting the joints, autophagy has a critical role in TNF-induced joint destruction (in murine experimental arthritis); TNF activates autophagy by induction of BECN1 and ATG7 and the conversion from MAP1LC3B-I to MAP1LC3B-II in osteoclasts, providing a mechanism for TNF-associated synovial inflammation and bone resorption.^92^ These findings suggest that TNF promotes autophagy-dependent osteoclast differentiation and survival, which is confirmed in human synovial fibroblasts.^93-95^

## The role of autophagy in infectious diseases

In the fight against pathogenic microorganisms, autophagy (this form of autophagy is called xenophagy) contributes to both innate immune defense and to development of adaptive immune memory, and excellent recent reviews have described these mechanisms in great detail.^96,97^ The role of autophagy in infectious diseases is apparent at the level of microbial handling by the innate immune system, as well as antigen processing and presentation of a wide variety of microorganisms ranging from viruses, to bacteria, fungi, and protozoa.

Within the context of innate host defense, autophagy provides the machinery for intracellular killing of microbes by means of phagophore engulfment and lysosomal fusion. Proper designation of microorganisms for autophagy processing is facilitated by protein adaptors such as SQSTM1/p62, NBR1 and CALCOCO2, that are activated downstream of microbial recognition by pattern-recognition receptors (TLR, NLR, RLR), providing anchors for phagophore assembly around the selected cargo.^67,98,99^ Several intracellular signaling pathways have been described to induce autophagy by PRRs. Activation of a TLR4-TICAM1 pathway has been reported to induce autophagy by Gram-negative bacteria such as *Pseudomonas aeruginosa*, and cleavage of TICAM1 by CASP1 inhibits autophagy.^100^ In addition, another important factor for TLR-induced autophagy is activation of the IRF8 transcription factor, which in turn activates genes involved in autophagosome formation.^101^ Induction of autophagy by NLR receptors is induced by the interaction with the peptidoglycans that activate a RIPK2/RIP2 (receptor-interacting serine-threonine kinase 2)-dependent signaling pathway.^102,103^ Furthermore, a very important process during the induction of autophagy is represented by the interaction with molecular sensors of metabolites. In this respect, during antimycobacterial responses induction of autophagy necessitates activation of AMPK that induces PPARGC1A (peroxisome proliferator-activated receptor gamma, coactivator 1 α), leading to activation of oxidative phosphorylation, inhibition of MTOR, and induction of autophagy.^104^ A similar dependency on MTOR inhibition and induction of ROS is required for induction of autophagy by *S. pneumonia*.^105^

Autophagy is also important for the induction of T cell responses, and recent studies have underlined its crucial role for lymphocyte survival and memory. During infection with lymphocytic choriomeningitis virus autophagy is not required for proliferation and CD8^+^ T-effector function, but is crucial for long-term cell survival and memory.^106^ A similar role of autophagy for CD8^+^ memory was reported during influenza and MCMV infections.^107^ During influenza infection, *Atg5* knockout mice display a defective lymphocyte survival and proliferation.^108^ Interestingly, chaperone-mediated autophagy regulates T cell responses through targeted degradation of negative regulators of T cell activation.^109^

At the level of autophagy-inflammation interaction, host defense mechanisms are directly influenced by the modulation of proinflammatory cytokines, as described above in detail. However, the host defense against various classes of microorganisms is not influenced to the same extent. One of the most important infections for which autophagy makes a major impact is tuberculosis. Several studies have demonstrated the crucial role of autophagy for elimination of mycobacteria.^20,110^ *M. tuberculosis* induces ubiquitin-mediated targeting in macrophages, resulting in delivery of the pathogen to autolysosomes. This response requires the autophagy receptors SQSTM1, CALCOCO2 and the DNA-responsive kinase TBK1 (TANK-binding kinase 1),^110^ while inhibition of MTOR through AMPK activation is a necessary change in metabolic status of the cell activating autophagy.^104^ In addition, antimycobacterial peptides are produced in a SQSTM1-dependent manner.^111,112^

Autophagy is also important in host defense against other intracellular pathogens such as *Toxoplasma gondii*,^113,114^ *Salmonella spp*.^115,116^ and *Listeria monocytogenes*.^117^ The role of autophagy for host defense against viruses strongly depends on the type of infection, and involves several mechanisms, especially those involved in autophagosome generation and maturation (as detailed in ref. 96), rather than inflammation. Autophagy is important for removal of herpes simplex virus-1^118^ and Rift valley virus,^119^ and restricts HIV infection by degrading the HIV1 transactivator Tat in CD4^+^ lymphocytes.^120^ In addition, autophagy not only contributes to the elimination of the virus, but also controls IL1B induction and aberrant induction of IL17-dependent lung pathology in respiratory syncytial virus infection.^121^ Autophagy also proved important for the host defense against the fungal pathogen *Cryptococcus neoformans*, through mechanisms involving the nonlytic exocytosis of the fungus, but also interfering with production of the inflammatory mediators IL6 and CXCL10/IP-10 (chemokine [C-X-C motif] ligand 10).^122^ In contrast, no effect of autophagy for host defense against *Candida albicans* has been demonstrated,^123,124^ despite the demonstration of CLEC7A/dectin-1-dependent accumulation of MAP1LC3B to phagosomes that increases in vitro killing of the fungus.^125^

Notably, pathogens have also evolved strategies to evade the immune response by taking advantage of the autophagy machinery. For instance, *M. tuberculosis* latently infects human immune cells by concealed residence in the autophagosome and by preventing lysosomal fusion.^20^ In addition, active mycobacterial infection counteracts autophagy through the induction of MIR30A that inhibits BECN1.^126^ *Salmonella* in turn attempts to inhibit autophagy and increase its survival by activating PTK2/focal adhesion kinase and MTOR.^127^ Furthermore, *Listeria monocytogenes* and *Shigella*, as well as viral pathogens such as HIV, have evolved different strategies to evade the autophagy machinery by inhibition of autophagosome formation or by modulation of positive (IFNA and IFNB-IL6 signaling) and negative upstream regulators (MTOR kinase signaling pathway) of autophagy.^128-130^

Genetic variants of autophagy genes have been investigated for their effects on antimicrobial immune responses, which revealed that the CD-associated *ATG16L1* polymorphism impairs defense mechanisms against Salmonella t*yphimurium* and increases susceptibility toward *Helicobacter pylori* infection.^53,131^ In addition, genetic variants of both *ATG16L1* and *IRGM* are associated with sepsis, potentially through modulation of immunoparalysis.^132,133^ However, in a large cohort no significant predisposition was observed for polymorphisms in autophagy genes to develop *M. tuberculosis* infections, although autophagy profoundly influences the host immune response against this pathogen.^134,135^ Similar findings have been reported for mucosal and systemic infections caused by the fungus *Candida* spp, indicating the redundant role of autophagy in anticandidal host defense.^136,137^

## The impact of autophagy-inflammation interplay on carcinogenesis

Both autophagy and inflammation are involved in several steps of carcinogenesis and cancer progression. Autophagy has been reported to have both antitumoral effects and tumor-promoting effects in cancer. The generally accepted explanation for this apparent ambiguous role of autophagy in cancer is that its role might differ in the different stages of carcinogenesis and in different tumor types and is related to changes in the tumor microenvironment and treatment. In addition, the dual role of autophagy in cancer might reflect the interplay between autophagy and other fundamental biological processes such as apoptosis, senescence, cell metabolism, and the immune system.^138,139^

In the early stages of cancer development, defects in autophagy machinery or inhibition of autophagy in premalignant cells might result in impaired degradation and removal of damaged organelles, unfolded proteins and ROS. These cytosolic components, which might accumulate in the cells, produced as a result of activation of oncogenic pathways result in DNA damage and maintain the carcinogenic phenotype.^140,141^ In mice, monoallelic deletion of haplo-insufficient *Becn1*, results in autophagy deficiency, leads to spontaneous development of malignant tumors, and stimulates development of hepatitis-B-induced premalignant liver lesions.^142^ Other studies indicate that BECN1 represents an important tumor suppressor and an increased BECN1 expression is associated with a favorable prognosis in several cancers.^143-146^ Moreover somatic mutations in *ATG* genes have been described in different malignant tumors.^147,148^ In addition, somatic mutations and/or genetic amplifications in *AKT1* and class I phosphoinositide 3-kinase (PI3K) and somatic or germ-line mutations leading to inactivation of the tumor suppressor gene *PTEN* are often found in malignant tumors.^149-153^As a result, signaling molecules upstream of MTOR such as AKT1 and class I PI3K are activated and exert inhibitory effects on autophagy in malignant transformed cells. AKT1 can inhibit autophagy either through activation of MTOR or through MTOR-independent mechanisms such as BECN1 phosphorylation and formation of an autophagy-inhibitory BECN1-YWHA/14–3–3-VIM/vimentin intermediate filament complex.^154^ The class III phosphatidylinositol 3-kinase (PtdIns3K) is an intracellular lipid kinase that has crucial functions in autophagosome formation, vesicular trafficking and endocytosis.^155^ In addition these lipid kinases represent key signaling molecules that play important roles in carcinogenesis. Three classes of phosphoinositide and phosphatidylinositol 3-kinases have been described according to their structure and substrate specificity. Of these, the functions of class I and of class III enzymes are best understood. Class III PtdIns3k phosphorylates phosphatidylinositol to form phosphatidylinositol-3-phosphate. The catalytic subunit of class III PtdIns3k, PIK3C3/Vps34, binds to other regulatory proteins, including ATG14, and contributes to the induction of autophagosome formation. In cancer, however, class I PI3Ks are often activated and therefore their role has been better explored as potential targets for therapy in malignant processes. In contrast to class III PtdIns3K, class I PI3K is activated downstream of receptor tyrosine kinases and G-protein coupled receptors and upon activation phosphorylates phosphatidylinositol 4,5-bisphosphate to phosphatidylinositol 3,4,5-trisphosphate. Through this, PI3K activates AKT and MTOR, which in turn promotes cell survival and proliferation and suppresses autophagy. ^156-158^ Altogether, these studies suggest that autophagy genes can act as tumor suppressor genes in several tumors, and suppressed or defective autophagy may play an important role in tumor initiation.

Conversely, in the more advanced stages of tumor development and progression, once the carcinogenic phenotype has been established, the cancer cells rely on autophagy to support the requirements for energy and provide substrates necessary for proliferation. This is especially important for surviving in an unfavorable microenvironment characterized by severe hypoxia and nutrient-deficient conditions. Therefore activation of autophagy in this context could be regarded as a survival factor, promoting cell proliferation and tumor progression. This is particularly relevant in the context of therapeutic response to cytotoxic agents and ionizing radiation. The strong induction of autophagy observed as a result of treatment with ionizing radiation or chemotherapy may contribute to cell survival and the acquired resistance to therapy in many cancer cells including breast cancer, pancreatic cancer, and malignant glioma.^159-162^ For this reason, inhibition of autophagy with chloroquine or hydroxychloroquine in combination with radiotherapy or chemotherapy has been investigated as a strategy to prevent therapy resistance and sensitize tumors to therapy.^163-167^ However, in the context of excessive activation autophagy may lead to cell death in cells lacking apoptotic machinery, in which the programmed cell death takes place preferentially through autophagic cell death.^168-172^ Several mechanisms have been postulated to explain these apparently conflicting roles of autophagy in tumorigenesis. These include involvement of autophagy in cell death and cell survival through an intricate interplay with apoptosis and necrosis by shaping the metabolic and oxidative stress environment of the cell, its role in quality control for the homeostasis of proteins and organelles, and its function in the modulation of tumor-targeted immune responses.^138,173^ However the exact regulatory mechanisms of this dynamic process are not completely understood and due to the length and the focus of this review this will not be further discussed here. We will focus instead on the role and the consequences of the interplay between autophagy and inflammation in cancer pathogenesis and treatment.

**Figure 4.**
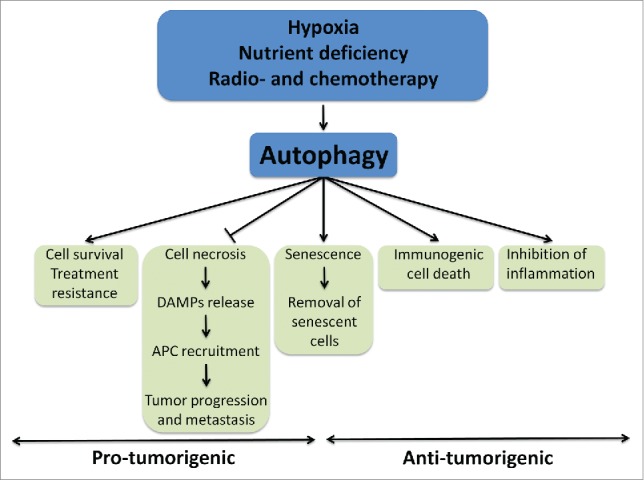
The role of crosstalk between autophagy and inflammation in cancer. Induction of autophagy is a double-edged sword with both protumorigenic and antitumorigenic effects. Processes downstream of autophagy that influence tumor progression and treatment resistance are comprised of cell survival pathways and recruitment of innate immune cells following release of DAMPs by necrotic tumor cells. Conversely, antitumorigenic signaling is evoked by autophagy-mediated immunogenic cell death and inhibition of tumor-associated inflammation. An outstanding example of the diverse and apparently opposite effects of autophagy on cancer initiation and progression is the induction of cellular senescence and subsequent removal of senescent cells; depending on the cellular context this is either pro- or anti-tumorigenic. APC, antigen-presenting cell.

The relationship between inflammation and cancer has long been recognized. Many chronic inflammatory conditions, such as inflammatory bowel disease, chronic hepatitis, or pancreatitis are clearly recognized as precursors of malignancies in the respective organs.^174-177^ Moreover, the immune system is the most important defense mechanism that identifies and eliminates malignant transformed cells and prevents tumor progression and metastasis. Autophagy has a modulatory role that shapes the interface between cancer and immune response, including effects on both tumor cells and immune cells, which subsequently influence the survival and function of, and interplay between, these cells and ultimately results in either tumor-promoting or tumor-suppressing effects (Fig. 4).

It is well known that tumor cells rely on aerobic glycolysis to support their energy requirements and to enable increased protein production (historically known as the Warburg effect).^178,179^ This mode of growth, however, requires an increased influx of glucose, which is often not available in parts of the tumor that have no or only limited access to (neo)vascularization. As described above, in conditions of glucose or amino acid deprivation and hypoxia autophagy is activated as an important survival mechanism. However, oncogenic events, such as mutations in the PI3K-AKT-MTOR signaling pathway, have anti-apoptotic effects and inhibit autophagy simultaneously. This impairs the activation of autophagy as a compensatory survival mechanism and renders the tumor cells prone to necrosis.^180^ In cancer, necrosis is seen as an unfavorable event because it results in release of highly inflammatory molecules, such as uric acid, ATP/UTP, HMGB1 (high mobility group box 1), and other damage-associated molecular patterns (DAMPs).^181-183^ All together, these inflammatory mediators and DAMPs promote and maintain malignant features such as cell survival and proliferation and contribute to recruitment of macrophages and other specialized antigen-presenting cells such as DCs.^184^ These cells in turn stimulate inflammation in the peritumoral stroma and induce a pro-tumorigenic environment that promotes angiogenesis, tumor progression, and metastasis.^180^

Although not an inflammatory mechanism itself, it is also important to underline that antigen recognition and presentation is the cornerstone of the specific immune responses against malignant cells. Hence, proper functioning of these processes heavily depends on the integrity of the autophagy machinery. It has for instance been shown that autophagy facilitates antigen presentation by tumor cells, such as human embryonic kidney cells (HEK293T) and melanoma cells, thereby facilitating the recognition of these cells by immune cells and their subsequent removal.^185^

In addition, autophagy induces cellular senescence, a physiological state of sustained cell cycle arrest characterized by a senescence-associated secretory phenotype. This consists of increased expression and secretion of several cytokines, chemokines, growth factors, and proteases in the senescent cells,^186^ which stimulate on the one hand the recruitment and activation of phagocytes responsible for the removal of these cells, and on the other hand contribute to maintenance of the senescent phenotype. Subsequently, failure to induce autophagic flux and immune-mediated senescence increases the susceptibility to hepatocellular carcinoma in a mouse model.^187^

The interplay between autophagy and inflammation is particularly interesting in the context of therapeutic implications, and the concerted targeting of these 2 processes can be explored for novel strategies. TNFSF10/TRAIL (tumor necrosis factor [ligand] superfamily, member 10) induces cell death in different cancer cells and therefore contributes to immune cell-mediated cytotoxicity.^188-190^ However, pharmacological treatment with recombinant ligands has been hampered by the occurrence of therapy resistance.^191^ Nevertheless, blocking autophagy in the FRO anaplastic thyroid cancer cell line with *ATG7* siRNA sensitizes the cells to TNFSF10-induced apoptosis, indicating that modulation of autophagy holds important therapeutic potential for anticancer treatment.^192^

Moreover, autophagy plays an important role in the response to chemotherapy of some tumors, in particular through influencing the immune response in the tumor microenvironment. Treatment with chemotherapy can induce immunogenic cell death.^193^ In this process, macrophages and DCs are attracted and recruited in the peritumoral stroma by molecules exposed at the cell surface or released in the tumor microenvironment as a result of apoptosis and necrosis following treatment with chemotherapeutic agents. DCs engulf the damaged tumor cells (remnants) and activate T-cells through antigen-presentation. These cells exert an antitumoral effect on the remaining unaffected cancer cells, thus enhancing the antitumoral effect of the chemotherapeutic agent. Michaud et al. have shown in cell lines and mouse models that autophagy is crucial for ATP release by the chemotherapy-induced dying tumor cells and for the induction of the antitumoral immunogenic response.^194^ In this study, therapy with methotrexate results in production of significantly lower amounts of ATP by autophagy-deficient tumors and a lack of antitumoral immunogenic responses. The immunogenicity of the autophagy-deficient cells could be restored after treatment with ARL67156, an inhibitor of ecto-ATPases that increase the extracellular ATP concentration, and after treatment with recombinant human IL1B, the production of which depends on the availability of ATP.^194^ Building upon these findings, Ko et al. showed that autophagy-dependent ATP release from stressed or dying tumors after radiotherapy contributes to lymphocyte infiltration in the tumor microenvironment and to the efficacy of radiotherapy in immunocompetent mice.^195^ Furthermore, Rao et al. report that non-small lung carcinoma tumors developing in autophagy-deficient KRAS; *atg5*^*fl/fl*^ mice have an accelerated growth compared to tumors developing in autophagy-competent KRAS; *Atg5*^*fl/+*^ animals. In this model, the autophagy deficient tumors also show increased infiltration with FOXP3^+^ regulatory T cells and increased expression of ENTPD1/CD39 ecto-ATPase. These effects can be reversed by pharmacological inhibition of ENTPD1, thus supporting the concept that autophagy deficiency contributes to carcinogenesis through inhibition of anticancer immunosurveillance.^196^

Therefore, these data indicate that the immunogenic responses and immunosurveillance against cancer are dependent on the interplay between autophagy and inflammation. In addition the autophagy status contributes significantly to tumorigenesis and to the efficacy of chemo- and radiotherapy.

## Conclusions and future perspectives

Autophagy influences several key components of the immune response, and thus plays a critical role in regulating inflammatory responses. It has even been suggested that autophagy evolved as a primordial host defense mechanism of eukaryotes.^3^ As evolution progressed, autophagy has intimately integrated with the other components of host defense, including the inflammatory reaction.

Despite the increasing knowledge about the role of autophagy in modulation of inflammation, this field is still only in its beginnings. Little is known about the involvement of the autophagy-related SQSTM1-like receptors for the modulation of inflammation, although some initial data have suggested important effects.^197,198^ The pathophysiological relevance of autophagy-inflammation interplay has not been studied yet in the context of colonization of mucosal membranes, and detection of tissue invasion. The influence of autophagy for directing polarization of adaptive immune responses (e.g., Th1 vs. Th2 vs. Th17) also warrants additional study in the coming years. In addition, the study of the balance between induction of autophagy and inhibition of inflammation is only in its beginning in the setting of understanding the pathophysiology of different types of cancer, and remains nearly untouched in autoimmune and autoinflammatory diseases. Understanding its role in tissue repair and remodeling is also an important challenge for the future.

Finally and most importantly, modulation of autophagy activity may represent a promising therapeutic approach for a wide range of inflammatory conditions. In this respect, a recent study reported the development of an autophagy-inducing peptide, Tat-BECN1, which decreased the replication of several human pathogens in vitro, and reduced mortality in mice infected with chikungunya or West Nile virus.^199^ However, at the same time, autophagy is a fundamental evolutionarily conserved process, and detailed knowledge about autophagy is necessary regarding its role in different diseases, cell types, and timelines, in order to avoid improper (or even dangerous) manipulation of a process fundamental for cell integrity. Nevertheless, future investigations are warranted in order to pursue novel autophagy-directed therapeutic approaches to effectively treat the ever-growing number of patients suffering from immune-mediated disorders.

## Abbreviations

*AMP*: adenosine monophosphate
*AMPK*: 5′ AMP-activated protein kinase
*ATG*: autophagy related
*ATP*: adenosine triphosphate
*BCG*: Bacillus Calmette-Guérin
*BECN1*: Beclin 1, autophagy related
*CD*: Crohn disease
*CGD*: chronic granulomatous disease
*CXCL10/IP-10*: chemokine (C-X-C motif) ligand 10
*DAMP*: danger-associated molecular pattern
*DC*: dendritic cell
*EAE*: experimental autoimmune encephalomyelitis
*GTP*: guanosine-5′-triphosphate
*IFN*: interferon
*IKBKB/IκB kinase*: inhibitor of kappa light polypeptide gene enhancer in B-cells, kinase beta
*IL*: interleukin
*IL1RN/ILR1A*: interleukin 1 receptor antagonist
*IRGM*: immunity-related GTPase family, M
*MAP1LC3B*: microtubule-associated protein 1 light chain 3 beta
*LAP*: LC3-associated phagocytosis
*LPS*: lipopolysaccharide
*mtDNA*: mitochondrial DNA
*MAPK*: mitogen-activated protein kinase
*MTOR*: mechanistic target of rapamycin (serine/threonine kinase)
*MYD88*: myeloid differentiation primary response 88
*NFKB*: nuclear factor of kappa light polypeptide gene enhancer in B-cells
*NLR*: NOD-like receptor
*NLRP3*: NLR family, pyrin domain containing 3
*NADPH*: nicotinamide adenine dinucleotide hydrogen phosphate
*NOD*: nucleotide-binding oligomerization domain
*PAMP*: pathogen-associated molecular pattern
*PRR*: pattern-recognition receptor
*PTEN*: phosphatase and tensin homolog
*PINK1*: PTEN-induced putative kinase 1
*RIPK2*: receptor-interacting serine-threonine kinase 2
*RA*: rheumatoid arthritis
*RLR*: RIG-I-like receptor
*ROS*: reactive oxygen species
*SLE*: systemic lupus erythematosus
*Th*: T helper
*TLR*: toll-like receptor
*TNF*: tumor necrosis factor
*TRAF*: TNF receptor-associated factor
*TNFSF10/TRAIL*: tumor necrosis factor (ligand) superfamily, member 10
*TICAM1/TRIF*: toll-like receptor adaptor molecule 1
*ULK1*: unc-51 like autophagy activating kinase 1.
